# Upfront Advanced Radiotherapy and New Drugs for NSCLC Patients with Synchronous Brain Metastases: Is the Juice Worth the Squeeze? A Real-World Analysis from Lombardy, Italy

**DOI:** 10.3390/cancers15041103

**Published:** 2023-02-09

**Authors:** Giulia Corrao, Matteo Franchi, Mattia Zaffaroni, Maria Giulia Vincini, Filippo de Marinis, Lorenzo Spaggiari, Roberto Orecchia, Giulia Marvaso, Barbara Alicja Jereczek-Fossa

**Affiliations:** 1 Division of Radiation Oncology, IEO—European Institute of Oncology, IRCCS, 20141 Milan, Italy; 2 National Centre for Healthcare Research and Pharmacoepidemiology, University of Milano-Bicocca, 20126 Milan, Italy; 3 Unit of Biostatistics, Epidemiology and Public Health, Department of Statistics and Quantitative Methods, University of Milano-Bicocca, 20126 Milan, Italy; 4 Division of Thoracic Oncology, IEO—European Institute of Oncology, IRCCS, 20141 Milan, Italy; 5 Department of Thoracic Surgery, IEO—European Institute of Oncology, IRCCS, 20141 Milan, Italy; 6 Department of Oncology and Hemato-Oncology, University of Milan, 20122 Milan, Italy; 7 Scientific Directorate, IEO—European Institute of Oncology, IRCCS, 20141 Milan, Italy

**Keywords:** NSCLC, brain metastases, stereotactic radiotherapy, TKI, immunotherapy, real-world analysis

## Abstract

**Simple Summary:**

This study aims to compare effectiveness and cost profile in non-small-cell lung cancer (NSCLC) patients harboring synchronous brain metastases (BMs) who received non-chemo first-line systemic therapy with or without advanced radiotherapy (aRT). A total of 177 lung cancer patients, of whom 58 were treated with systemic treatment (either TKIs or pembrolizumab) plus aRT (STRT) and 119 with systemic treatment alone, were selected. The addition of aRT to systemic treatment was associated with a significantly better OS (*p* = 0.020) and PFS (*p* = 0.041) than systemic therapy alone. The incremental cost-effectiveness ratio (ICER) value indicated an average cost of €3792 for each month of survival after STRT and confirmed clinical effectiveness but higher healthcare costs. This real-world study suggests that upfront aRT in this setting represents a valid treatment strategy, boosting the efficacy of emerging drug classes with sustainable costs for the health service.

**Abstract:**

Aim: Healthcare administrative databases represent a valuable source for real-life data analysis. The primary aim of this study is to compare effectiveness and cost profile in non-small-cell lung cancer (NSCLC) patients harboring synchronous brain metastases (BMs) who received non-chemo first-line systemic therapy with or without advanced radiotherapy (aRT). Methods: Diagnostic ICD-9-CM codes were used for identifying all patients with a new diagnosis of lung cancer between 2012 and 2019. Among these, patients who had started a first-line systemic treatment with either TKIs or pembrolizumab, alone or in combination with intensity-modulated or stereotactic RT, were selected. Clinical outcomes investigated included overall survival (OS), progression-free survival (PFS), and time-to-treatment failure (TTF). The cost outcome was defined as the average per capita cumulative healthcare direct costs of the treatment, including all inpatient and outpatient costs. Results: The final cohort included 177 patients, of whom 58 were treated with systemic treatment plus aRT (STRT) and 119 with systemic treatment alone. The addition of aRT to systemic treatment was associated with a significantly better OS (*p* = 0.020) and PFS (*p* = 0.041) than systemic therapy alone. The ICER (incremental cost-effectiveness ratio) value indicated an average cost of €3792 for each month of survival after STRT treatment and confirmed clinical effectiveness but higher healthcare costs. Conclusions: This real-world study suggests that upfront aRT for NCLSC patients with synchronous BMs represents a valid treatment strategy, boosting the efficacy of novel and emerging drug classes with sustainable costs for the health service. Translational relevance: The present real-world study reports that the use of upfront advanced radiotherapyaRT and new-generation systemic agents, such as TKIs and pembrolizumab, may have higher oncological control and an improved cost-effectiveness profile than the use of new-generation systemic agents alone in NCLSC patients with synchronous brain metastases. Acquired evidence can also be used to inform policymakers that adding advanced radiotherapy results is a sustainable cost for the health service. Since approximately 50% of patients do not meet RCT inclusion criteria, a significant proportion of them is receiving treatment that is not evidence-informed; therefore, these results warrant further studies to identify the best radiotherapy timing and possible dose escalation approaches to improving treatment efficacy in patient subgroups not typically represented in randomized controlled trials.

## 1. Introduction

Despite therapeutic advances, lung cancer still accounts for 18% of all cancer-associated mortality [1], with 70–80% of patients presenting with metastatic and/or unresectable disease [2]. Current therapeutic strategies are tailored to particular genetic variants. Target therapies, such as tyrosine-kinase inhibitors (TKIs), improve prognosis of patients with common variants [3], while immunotherapy is the primary first- and second-line treatment for patients without these mutations [3]. 

Brain metastases occur in up to 25% of patients with non-small-cell lung cancer (NSCLC) at baseline [4,5]. While new treatments for advanced NSCLC (aNSCLC) are continuously developed, and outcomes are improving, patient prognosis remains poor, and a median survival of 12–15 months is currently reported [6]. This is the result of unmet needs that still persist. In particular, the effective management and treatment of brain metastases, given their relatively high incidence in aNSCLC patients, still represents a major challenge.

Stereotactic radiotherapy (STRT) is a ground-breaking technique that is strongly recommended when feasible [7]. New-generation systemic agents, including TKIs and immunotherapy, can achieve oncological benefits by exploiting the STRT intracranial antitumor activity [8,9,10,11]. Current guidelines from the American Society of Clinical Oncology, Society for Neuro-Oncology, and the American Society for Radiation Oncology indicate that new-generation drugs can defer local treatment of asymptomatic brain metastases until eventual progression; nevertheless, the strength of this recommendation remains weak [7].

Historically, only a small fraction of NSCLC patients with brain metastases at baseline have been offered upfront local treatments [12]. While several non-randomized controlled trials (RCT) have assessed the intracranial efficacy of immunotherapy for aNSCLC patients [13,14], the ability of STRT in combination with these new-generation systemic agents to control brain metastases remains unknown. In a rapidly changing treatment landscape, real-world data could offer important insights into the best strategies for managing this disease. 

Thus, a large population-based observational study was performed to compare the effectiveness of first-line systemic therapy with or without STRT on brain metastases in aNSCLC patients not receiving chemotherapeutic agents. The cost-effectiveness of such treatment patterns was a particular focus of this study.

## 2. Methods

### 2.1. Data Sources

The data used in the present study were retrieved from the healthcare utilization databases of Lombardy, Italy, a region with 10 million residents that accounts for about 16% of the country’s population. Through the National Health Service (NHS), Italian citizens have equal access to health care services that are considered essential, including those involved in cancer care. An automated system of healthcare utilization (HCU) databases allows each Italian region to locally manage NHS data. This includes information on residents who receive NHS assistance (NHS beneficiaries), diagnosis at discharge from public or private hospitals, outpatient drug prescriptions, specialist visits, and reimbursable diagnostic examinations. The cost of every service provided to an NHS beneficiary and reimbursed to a healthcare provider (i.e., direct healthcare costs for the Regional Health Authority) is also routinely recorded. A unique individual identification code is used to link the databases. To maintain privacy, each identification code is anonymized so that individuals can only be identified by the Regional Health Authority upon request from judicial authorities. Full details of the procedures are reported elsewhere [15]. Specific diagnostic and therapeutic codes used for the current study are reported in Table S1.

### 2.2. Cohort Selection

The selection criteria for the study cohort are detailed in a recent publication investigating the clinical and economic outcomes of aNSCLC patients treated with first-line TKIs or immunotherapy (pembrolizumab) in the Lombardy Region [16]. In brief, the target cohort included 37,562 Lombardy residents, all of whom were RHS beneficiaries, aged ≥18 years, and who had received a new lung cancer diagnosis between 2012 to 2019. Patients who had started a first-line systemic treatment with either TKIs or pembrolizumab, the only approved and reimbursable first-line systemic agents at the time, before June 30, 2020, were identified. The first-line treatment start date was defined as the “index date”. Only patients with a diagnosis of brain metastases between 30 days before and 90 days after the index date were included in the study cohort. Patients were classified as exposed to both systemic therapy and radiotherapy (STRT) or to systemic therapy only (STO) based on whether they did or did not receive stereotactic radiotherapy or intensity-modulated radiotherapy (advanced radiotherapy) during the period from 30 days before to 90 days after the index date. As patients treated with standard whole-brain 3D conformal radiotherapy are expected to have different clinical characteristics than those who received advanced radiotherapy, and as these characteristics are not measurable in a study based on HCU data, we preferred to exclude patients on standard radiotherapy. 

Because exposure to advanced radiotherapy was measured up to three months after the start of systemic therapy, patients who did not survive the first three months after treatment initiation were excluded. 

In order to avoid immortal-time bias [17], each member of the selected cohort accumulated person-years of follow-up starting three months after the index date until the earliest of two dates: outcome occurrence or the study endpoint (set on 31 December 2020).

### 2.3. Baseline Characteristics

Baseline covariates included sex, age at index date, and surgery between the first lung cancer diagnosis and the index date. The Cancer Multimorbidity Score (CMS), a recently developed score used to predict mortality in elderly cancer patients [18], was calculated during the two years before the index date. Conditions included in the CMS are reported in Table S2.

### 2.4. Outcomes

Clinical outcomes included (1) overall survival (OS), defined as the time between index date and death from any cause (the main outcome of interest), or censoring (migration to another region or study endpoint), whichever came first; (2) progression-free survival (PFS), defined as the time between the index date and a change in systemic treatment (i.e., a secondary outcome substituting for disease progression), or censoring (death for any cause, migration to another region or study endpoint), whichever came first (A further definition of PFS included the time to a change in systemic treatment, death from any cause, or the start of RT ≥ 90 days from the first-line systemic treatment, which could substitute for disease progression.); (3) time-to-treatment failure (TTF), defined as the time between the index date and treatment discontinuation for any cause (i.e., another secondary outcome substituting for treatment failure), or censoring (migration to another region or study endpoint), whichever came first.

The cost outcome was defined as the average per capita cumulative healthcare direct costs sustained by the RHS for the treatment of patients included in the study cohort, including all inpatient and outpatient costs (for any treatment provided by the RHS, not just for cancer care) from the index date to the earliest date between death or censoring.

## 3. Statistical Analyses

Between treatment arms, differences in baseline characteristics were tested by the Chi-square or Fisher exact test. The Kaplan–Meier estimator was used for measuring cumulative OS, PFS, and TTF. The Cox proportional hazard regression model was used to estimate the hazard ratio (HR) and its 95% confidence interval (CI), for the association between exposure to advanced radiotherapy and clinical outcomes. As exposure may change over time, assessment of its effect requires consideration of its cumulative and varying nature. This was performed by fitting the Cox model, including dummy factors for exposure categories expressed as time-dependent covariates. Adjustments were made for sex, age categories, type of first-line systemic treatment, and CMS. 

Cumulative healthcare costs (CHC) by treatment type were calculated using the Bang and Tsiatis estimator [19], which takes censored cost data into account. CHC was calculated for each patient by summing direct costs sustained by the NHS. The cost-effectiveness profile was assessed by dividing the between-arm differences into healthcare costs and health-related outcomes determined using the restricted mean survival time [20]. The non-parametric bootstrap method based on 1000 re-samples [21] was used to explore the uncertainty in cost-effectiveness estimates [22]. All analyses were performed using SAS 9.4 (Cary, NC, USA). Statistical significance was set at the 0.05 level. All *p*-values were two-sided.

### Ethical Issues

The present study was approved by the Ethical Committee of the European Institute of Oncology (IRCCS Istituto Europeo di Oncologia, Milan, Italy) and the IRCCS Cardiological Centre “Monzino” of Milan, Italy (UID 2819).

## 4. Results

### 4.1. Patients

**Figure 1:**
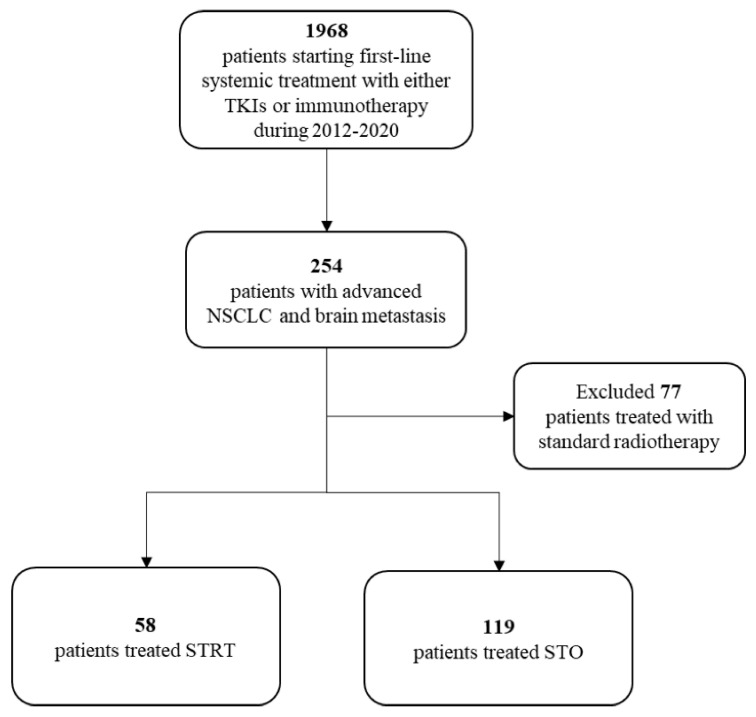
Flow-chart of cohort selection. List of abbreviations: NSCLC = non-small-cell lung cancer; STRT = systemic therapy and radiotherapy; STO = systemic therapy only; TKIs = tyrosine kinase inhibitor.

Of the 1968 aNSCLC patients who started a first-line systemic treatment with either TKIs or pembrolizumab during the study period, 254 had signs of brain metastases within 120 days around the diagnosis. Of these, 77 patients received standard RT and were therefore excluded from the analysis. The final cohort included 177 patients, of whom 58 were treated with STRT and 119 were treated with STO (Figure 1). 

Baseline characteristics of the patients are shown in Table 1. Patients receiving STRT were more likely to be male than those receiving only systemic therapy (53.5% vs. 40.3%, respectively; *p* = 0.001) and were more likely to be treated with first-line pembrolizumab (51.7% vs. 24.4%, respectively; *p* < 0.001). No differences were observed in surgery, CMS, and selected comorbidities, including arterial hypertension, diabetes mellitus, hyperlipidaemia, and renal diseases, between the treatment groups.

### 4.2. Survival Analysis

**Figure 2:**
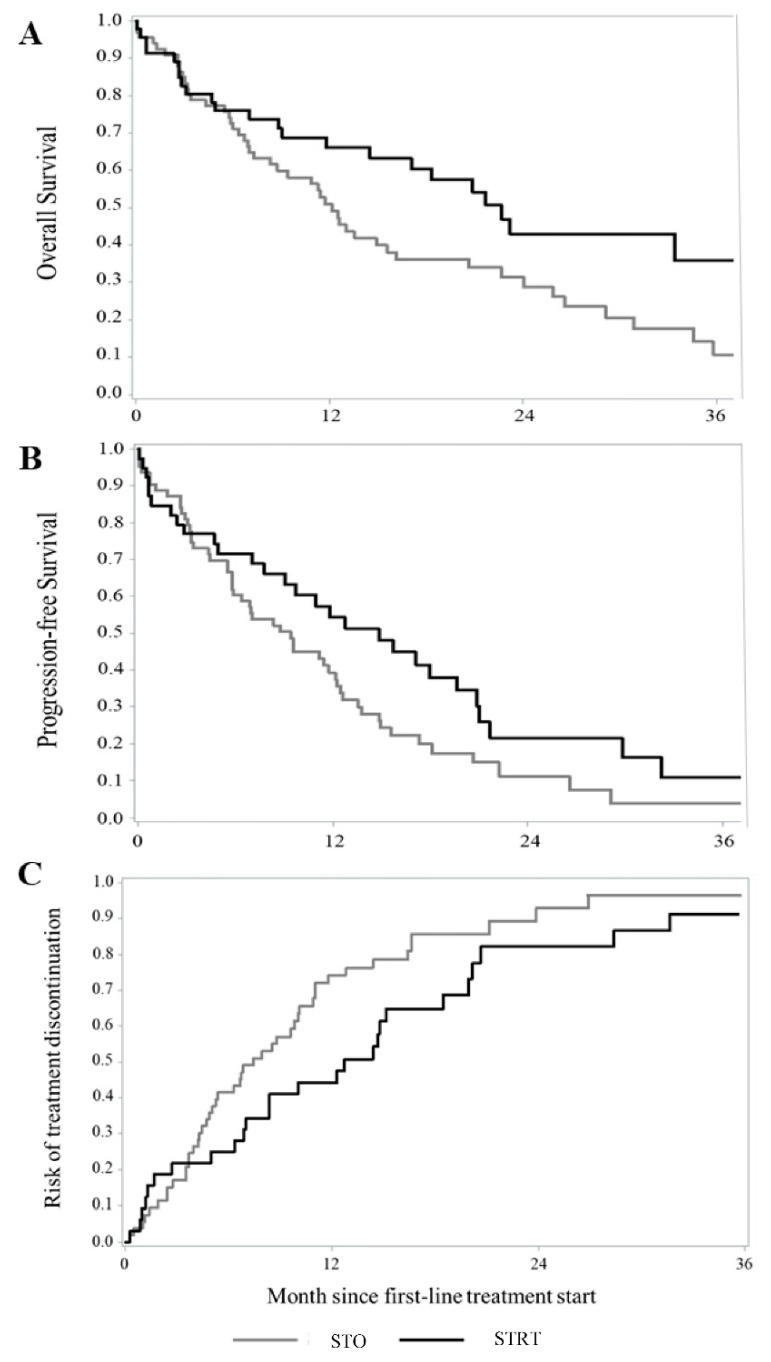
Kaplan–Meier estimates of overall survival (**A**), progression-free survival (**B**), and time-to-treatment failure (**C**) among patients with advanced NSCLC and synchronous brain metastasis treated with systemic therapy and advanced radiotherapy (STRT) or with systemic therapy only (STO). Footnote: In order to avoid immortal-time bias, in this analysis, only patients who survived to the first three months of follow-up were included. For each patient, follow-up starts three months after the start of first-line systemic therapy.

Kaplan–Meier estimates of survival are shown in Figure 2A–C. Of patients who survived the first three months of follow-up, 46 and 66 were treated with STRT or systemic therapy alone, respectively. The addition of radiotherapy to systemic treatment was associated with a significantly better OS (median OS 22.6 vs. 12.1 months, respectively; *p* = 0.020) and PFS (median PFS 14.8 vs. 9.4 months, respectively; *p* = 0.041), than systemic therapy alone, while differences in the TTF were not significant (median TTF 12.7 vs. 7.4 months, respectively; *p* = 0.065). 

A significantly higher reduction in the risk of death was observed in the 58 patients treated with STRT than in the 119 patients receiving systemic therapy alone, with a corresponding HR of death of 0.53 (95% CI: 0.35–0.79). STRT was also significantly associated with a longer PFS, with corresponding HRs of 0.59 (0.40–0.86) and 0.60 (0.42–0.87), depending on whether the primary or secondary PFS definition was used. A significant reduction in the risk of treatment failure was observed in STRT-treated patients (HR 0.59, 0.41–0.86) (Table 2). 

### 4.3. Healthcare Costs

**Figure 3:**
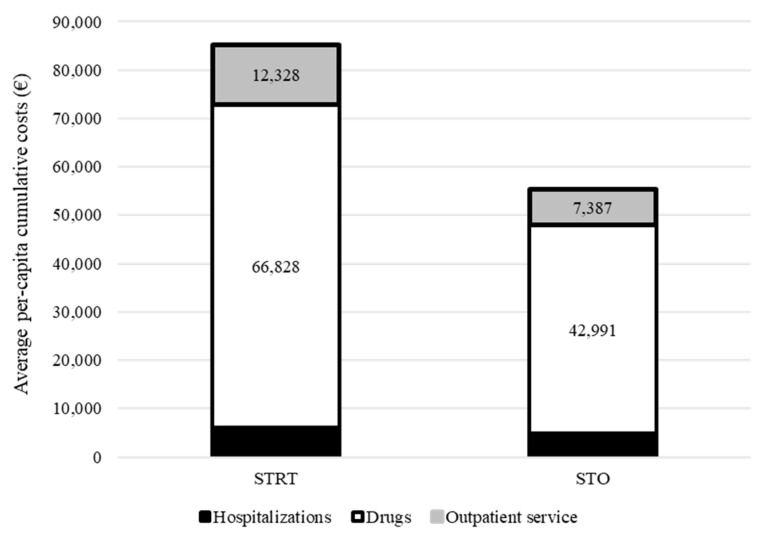
Comparison between cumulative per capita healthcare costs sustained by the National Health Service for taking care of patients with advanced NSCLC and synchronous brain metastasis treated with systemic therapy and advanced radiotherapy (STRT) or with systemic therapy only (STO). Footnotes. In this analysis, only 46 patients treated with STRT and 66 patients treated with STO who survived to the first three months of follow-up were included. For each patient, follow-up starts three months after the start of first-line systemic therapy.

**Figure 4:**
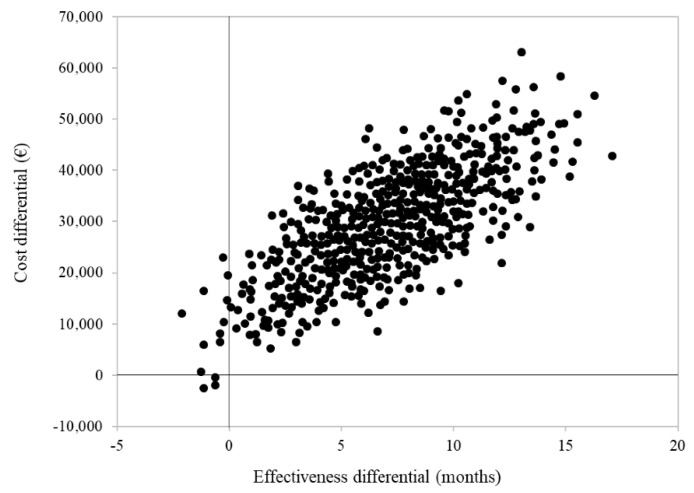
Cost-effectiveness analysis comparing patients with advanced NSCLC and synchronous brain metastasis treated with systemic therapy and advanced radiotherapy (STRT) or with systemic therapy only (STO). Footnotes. In order to avoid immortal-time bias, this analysis only included 46 patients treated with STRT and 66 patients treated with STO who survived to the first three months of follow-up. For each patient, follow-up starts three months after the start of first-line systemic therapy.

Cumulative healthcare costs by treatment group are shown in Figure 3. Overall, EUR 85,273 and EUR 55,316 were spent, on average, for each patient treated with STRT or systemic therapy alone, respectively. The cost-effectiveness profile is shown in Figure 4. The ICER (incremental cost-effectiveness ratio) value indicated an average cost of EUR 3792 for each month of survival after STRT treatment and confirmed clinical effectiveness (i.e., longer survival for patients on STRT therapy than systemic therapy alone), but higher healthcare costs.

## 5. Discussion

This population-based retrospective real-world study provides evidence that among aNSCLC patients with signs of brain metastases at baseline, the joint use of advanced radiotherapy and new-generation systemic agents, such as TKIs and pembrolizumab, may have higher oncological control and an improved cost-effectiveness profile than the use of new-generation systemic agents alone. Patients on both advanced radiotherapy and systemic therapy had a significant risk reduction of death, disease progression, and treatment failure of 47%, 41%, and 41%, respectively. To the best of our knowledge, this is the first study to evaluate the impact of innovative systemic treatment with intracranial activity on a regionally representative cohort of naïve aNSCLC patients with brain metastases. The results can also be used to inform policymakers that the additional healthcare cost incurred by adding advanced radiotherapy to new-generation systemic therapy was EUR 45,000 per year of life gained. This is lower than the willingness-to-pay thresholds of EUR 50–100,000 per year of life gained that are frequently used by Western countries [23,24]. 

The development of new drug classes, such as immunotherapy and TKIs, that are able to cross the blood-brain barrier, has changed the therapeutic regimen and allowed for local treatment deferral. Indeed, results from the KEYNOTE-024 [25] and KEYNOTE-042 [26] studies show that the first-line drug, pembrolizumab, is commonly administered without any patient-specific limitations. However, assuming that approximately 50% of patients do not meet RCT inclusion criteria as a result of brain metastases and performance status, then a significant proportion is receiving treatment that is not evidence-informed [5,12]. A recent phase two trial by Goldberg et al. investigated pembrolizumab intracranial activity in NSCLC patients with naïve or progressive brain metastasis [14]. Interestingly, treatment was only found to benefit patients with PD-L1-expressing tumors (>1%), suggesting that upfront RT avoidance or delay may be justifiable for a selected cohort that is receiving immunotherapy alone. Moreover, a recent study by Hendriks et al. investigated the role of immune checkpoint inhibitors (ICI) in a cohort of 1052 aNSCLC patients with synchronous brain metastases [27] and found that upfront local radiation treatment resulted in a significantly better outcome than no radiation (*p* < 0.001), including improved PFS and OS. 

While TKI represents the current elective treatment choice for driver mutations [28,29], these mutations usually correlate with a higher incidence of brain metastases, reaching up to 20–30% of some patient populations, particularly those with ALK translocation [30]. Several studies have investigated the efficacy and timing of local intracranial treatments in this setting, but no consensus has yet been reached [31,32]. Although TKIs are active in the central nervous system, providing patients up to 10 months PFS without irradiation [33,34], it is speculated that this approach could allow for local treatment deferral. This is supported with results from the current study, indicating that only 58 of 177 (33%) eligible patients received upfront advanced radiotherapy. A systematic review/meta-analysis by Soon et al. similarly concluded that upfront radiotherapy improves OS in EGFR-mutant aNSCLC patients receiving TKI [35]. A subsequent study by Magnuson et al. also reported that radiotherapy deferral (including both standard and intensity-modulated radiotherapy) results in lower survival rates [36]. 

There are some important considerations for defining the best treatment sequence for patients in the current study. The first consideration is the number of brain metastases at diagnosis. Miyawaki et al. reported that patients with 1–4 lesions who received upfront local treatment had higher survival rates than those who received upfront TKI [37]. Chang et al. also reported better survival outcomes after local therapies for patients with three or fewer lesions [38]. The second consideration is the radiosensitising potential of some TKIs. As a result of this, TKIs can have a potentially proapoptotic and antiproliferative effect when used in combination with radiotherapy [39,40]. The effect of TKIs may also be increased because of radiotherapy-induced damage to the blood-brain barrier that occurs when endothelial cells die [41,42]. The third consideration is the distinct intracranial activity of each TKI. Gefitinib (n = 106) and afatinib (n = 35) were the most commonly used TKIs in the current study [43,44,45,46]. Despite their low cerebrospinal fluid penetration rate, both these first-generation EGFR TKIs are effective at managing brain metastases [47]. A recent global GioTag study reported a median TTF of 22.2 months for patients with naïve brain metastases who received sequential afatinib plus osimertinib treatment [48]. In the present study, a TTF of 13 months was observed for patients treated with advanced radiotherapy and first-line TKI/IO, while radiotherapy-free patients experienced a TTF of 7 months. Other less-represented TKIs in this cohort, osimertinib (n = 11), crizotinib (n = 7), and alectinib (n = 6), demonstrated a different level of control on central nervous system spread. While osimertinib [13] and alectinib [49] show a high local control of intracranial disease, crizotinib is associated with a low cerebrospinal fluid-to-plasma ratio [50].

The current study has some limitations. First, despite the fact that this study was population-based, ideally including all patients with lung cancer undergoing first-line new-generation systemic agents, the strict eligibility criteria lead to a relatively small sample size and consequent power limitations. Effectively, a posthoc power analysis revealed that the current sample size allows for appreciating at least a 54% reduction in outcome occurrence (e.g., from 50% to 23% in OS by adding advanced radiotherapy to systemic therapy only) by accepting a two-sided 5% first-type error. Second, the data did not allow for direct identification of NSCLC patients (diagnostic ICD-9-CM codes do not distinguish between cancer sub-types), so the target NSCLC patient population from which the cohort was generated remains unknown. In addition, the percentage of aNSCLC patients who started a first-line systemic treatment was unknown, because these data could not be linked with other sources, such as histology data. Third, ICD-9-CM diagnostic codes only refer to inpatient diagnoses, and those provided in an outpatient setting are not available. In addition, the patients could not be stratified by the number of metastases, a well-established determinant of overall cancer outcomes. Finally, the study lacked comprehensive clinical, biological, and radiotherapy-related data, such as dose and fractionation scheme or intent (palliative, ablative etc.). As in any observational study, patients were not randomly allocated to systemic therapy alone or in combination with radiotherapy, so the results may be affected by confounding. The association between exposure to radiotherapy and clinical outcomes may be the result of unmeasured factors that influenced both the likelihood of receiving radiotherapy and the probability of experiencing the clinical outcomes of interest.

## 6. Conclusions

This study suggests that upfront advanced radiotherapy for NCLSC patients with synchronous brain metastases represents a valid treatment strategy, boosting the efficacy of novel and emerging drug classes with sustainable costs for the health service. These results could inform further trials or boost the implementation of additional clinical data in the regional healthcare utilization databases in order to identify the best radiotherapy timing and possible dose escalation approaches to improving treatment efficacy in patient subgroups not typically represented in RTCs.

## Figures and Tables

**Table 1:** Baseline characteristics of 177 patients with advanced NSCLC and brain metastasis included in the study cohort.

	STRT N = 58	STO N = 119	*p*-Value
Sex			
Male	31 (53.5)	48 (40.3)	0.010
Female	27 (46.5)	71 (59.7)	
Age categories (years)			
<60	20 (34.5)	30 (25.2)	0.415
60–69	19 (32.8)	35 (29.4)	
70–79	13 (22.4)	36 (30.3)	
≥80	6 (10.3)	18 (15.1)	
First-line systemic treatment			
Pembrolizumab	30 (51.7)	29 (24.4)	<0.001
Tyrosine kinase inhibitors	28 (48.3)	90 (75.6)	
Surgery			
Yes	7 (12.1)	9 (7.6)	0.327
No	51 (87.9)	110 (92.4)	
Cancer multimorbidity score			
0–4	22 (37.9)	34 (28.6)	0.465
5–9	25 (43.1)	66 (55.5)	
10–14	9 (15.5)	15 (12.6)	
≥15	2 (3.5)	4 (3.3)	
Comorbidities			
Hypertension	13 (22.4)	37 (31.1)	0.229
Diabetes	5 (8.6)	13 (10.9)	0.634
Hyperlipidaemia	15 (25.9)	33 (27.7)	0.793
Renal diseases	0 (0)	3 (2.5)	0.552

List of abbreviations: NSCLC = non-small-cell lung cancer; STRT = systemic therapy and radiotherapy; STO = systemic therapy only.

**Table 2:** Comparison of overall survival, progression-free survival, and time-to-treatment failure between 58 patients with advanced NSCLC and brain metastases treated with systemic therapy and radiotherapy and 119 patients treated with systemic treatment only.

	Overall Survival		Progression-Free Survival		Time-to-Treatment Failure	
	N (%) of events	HR * (95% CI)	N (%) of events	HR * (95% CI)	N (%) of events	HR * (95% CI)
First-line treatment						
STO	103 (86.6)	Reference	109 (91.6)	Reference	113 (95.0)	Reference
STRT	36 (62.1)	0.53 (0.35–0.79)	44 (75.9)	0.59 (0.40–0.86)	53 (91.4)	0.59 (0.41–0.86)

* Hazard ratio adjusted for sex, age categories, cancer multimorbidity score, and type of first-line systemic treatment. List of abbreviations: HR = hazard ratio; NSCLC = non-small-cell lung cancer; STRT = systemic therapy and radiotherapy; STO = systemic therapy only.

## Data Availability

The data that support the findings of this study are available from the corresponding author, M.Z., upon reasonable request.

## Supplementary Materials

The following supporting information can be downloaded at: https://www.mdpi.com/article/10.3390/cancers15041103/s1, Table S1: ICD-9-CM codes of diagnosis/procedures, ATC codes of drugs and Regional codes of outpatient’s services that will be used for the current study; Table S2: List of conditions and weights assigned to the Cancer Multimorbidity Score.

## Abbreviations

aNSCLC	Advanced Non-Small-Cell Lung Cancer
ASCO	American Society of Clinical Oncology
ASTRO	American Society of Radiation Oncology
CHC	Cumulative Healtcare Cost
CMS	Cancer Multimorbidity Score
HCU	Healthcare Utilization
HR	Hazard Ratio
ICER	Incremental Cost-Effectiveness Ratio
NHS	National Health Service
NSCLC	Non-small cell lung cancer
OS	Overall Survival
PFS	Progession Free Survival
RCT	Randomized Controlled Trial
SNO	Society of Neuro-Oncology
STO	Systemic Treatment Only
STRT	Systemic Treatment and Radiotherapy
TKI	Tyrosine Kinase Inhibitor
TTF	Time to Treatment Failure
