# Supplementary material for: Upfront Advanced Radiotherapy and New Drugs for NSCLC Patients with Synchronous Brain Metastases: Is the Juice Worth the Squeeze? A Real-World Analysis from Lombardy, Italy
Source: Cancers (Basel). 2023 Feb 9;15(4):1103. doi: 10.3390/cancers15041103 (PMC9953825; doi:10.3390/cancers15041103)
Supplement: Supplementary file 1 [file cancers-15-01103-s001.zip › cancers-2139310-supplementary.pdf]

Table S1. ICD-9-CM codes of diagnosis/procedures, ATC codes of drugs and Regional codes of outpatient's services that will be used for the current study.

Diagnosis (ICD-9-CM codes)	
Lung cancer	162* (except 162.0)
Chemotherapy	V58.1
Radiotherapy	V58.0
Brain metastasis	198.3
Procedures (ICD-9-CM codes)	
Lung surgery	
Other local excision or destruction of lesion or tissue of lung	32.29
Segmental resection of lung	32.3
Lobectomy of lung	32.4
Complete pneumonectomy	32.5
Radical dissection of thoracic structures	32.6
Other excision of lung	32.9
Chemotherapy/antineoplastic biological agents	99.25, 99.28
Radiotherapy	92.2, 92.3
Drugs (ATC codes)	
Immunotherapy	
Nivolumab	L01XC17
Pembrolizumab	L01XC18
Atezolizumab	L01XC32
Tyrosine kinase inhibitors (TKI)	
Erlotinib	L01XE03
Gefitinib	L01XE02
Afatinib	L01XE13
Dacomitinib	L01EB07
Osimertinib	L01EB04
Crizotinib	L01ED01
Alectinib	L01ED03
Brigatinib	L01ED04
Trametinib	L01EE01
Dabrafenib	L01EC02
Lorlatinib	L01ED05
Chemotherapy	
Cisplatin	L01XA01
Carboplatin	L01XA02
Docetaxel	L01CD02
Pemetrexed	L01BA04

Paclitaxel	L01CD01
Vinorelbin	L01CA04
Gemcitabin	L01BC05
Outpatients service (Regional codes)	
Chemotherapy with administration of high-cost drugs (file F 5 e 3)	MAC01
Chemotherapy without administration of high-cost drugs (file F 5 e 3)	MAC02
Anticancer therapy with administration of oral drug or IM	MAC04
Injection or infusion of chemotherapy drugs	99.25
Standard radiotherapy (RT)	92.24.3, 92.24.5, 92.29.F, 92.29.G, 92.29.J, 92.29.H
Stereotactic radiotherapy (SRT) or intensity modulated radiotherapy (IMRT)	92.24.6, 92.24.7, 92.24.8, 92.29K, 92.29.M, 92.29.L

Table S2. List of conditions and weights assigned to the Cancer Multimorbidity Score.

Disease/condition	Weight
Liver cirrhosis and other liver chronic diseases	8
Coagulation defects	5
Other diseases of the respiratory system	5
Insulin therapy	4
Dementia/Alzheimer	4
Epilepsy and recurrent seizures	3
Cystic fibrosis	3
Chronic pain	3
Psychosis	3
Chronic obstructive pulmonary disease	2
Other kidney disorders	2
Cerebrovascular diseases	2
Vascular diseases	2
Anaemias	2
Other diseases of the digestive system	2
Diabetes without insulin therapy	2
Disorders of fluid, electrolyte, and acid-base balance	2
Parkinson's disease	2
Corticosteroids	2
Chronic kidney disease (with or without dialysis)	2
Infectious and parasitic diseases (HIV infections, tuberculosis or other infectious and parasitic diseases)	2
Heart failure	1
Multiple sclerosis or other diseases of the nervous system and sense organs	1
Depression	1
Autoimmune disease (Rheumatoid arthritis, Rheumatoid psoriasis, Anchylosing spondylitis, Systemic sclerosis, Systemic lupus erythematosus) or other diseases of the musculoskeletal system and connective tissue	1
Other diseases of the circulatory system	1
Oral anticoagulant agents	1

Chronic respiratory disease only tracked by drug therapy	1
Arrhythmia	1
Gout	1
